# Enhancing Fluoride Mediated Dentine Sensitivity Relief through Functionalised Tricalcium Phosphate Activity

**DOI:** 10.1155/2015/905019

**Published:** 2015-04-02

**Authors:** Steven J. Naoum, Andrea Lenard, F. Elizabeth Martin, Ayman Ellakwa

**Affiliations:** ^1^Faculty of Dentistry, The University of Sydney, NSW, Australia; ^2^Westmead Centre for Oral Health, Westmead Hospital, Sydney, NSW 1245, Australia

## Abstract

*Background.* To assess the clinical efficacy of a dentifrice containing fluoride and functionalised tricalcium phosphate (fTCP) in reducing dentine sensitivity. *Methods.* A 10-week parallel blind randomised control trial was conducted. Subjects were assigned to one of four groups and instructed to brush twice daily: A: Colgate Cavity Protection (1000 ppmF-MFP); B: Sensodyne Total Care (1000 ppmF-NaF + 19300 ppmK^+^-KNO3); C: Clinpro Tooth Crème (950 ppmF-NaF + fTCP); and D: Clinpro Tooth Crème (brushing + additional topical application). Seventy-one patients were assessed at baseline, 6 weeks, and 10 weeks for cold, tactile, and hypertonic sensitivity using the NRS-11 pain rating scale. A combined modalities sensitivity score (CMS) was calculated. *Results.* At 6 weeks, patients reported the following reduction in CMS: A (20%); B (30%); C (42%); D (52%). At 10 weeks, patients reported the following reduction in CMS: A (18%), B (40%), C (24%), and D (54%). The only CMS comparisons to show a significant difference (*P* < 0.05) were between Groups A and D (6 and 10 weeks). *Conclusions.* Addition of fTCP to a dentifrice enhances the ability of dentifrice fluoride in reducing dentine sensitivity. Using Clinpro Tooth Crème twice daily for brushing can be as effective to reduce dentine sensitivity as twice daily brushing using Sensodyne Total Care. However, additional nightly topical application of fTCP, in addition to twice daily brushing, showed an enhanced reduction in dentine sensitivity.

## 1. Introduction

Dentine sensitivity is defined as short, sharp pain from exposed dentine in response to thermal, tactile, osmotic, or chemical stimuli that cannot be ascribed to any other dental defect or disease [1]. Dentine sensitivity affects 4–74% of the population [2]. Clinically the presence of dentine sensitivity creates challenges for both patients and dental practitioners; in addition to causing patient discomfort, dentine sensitivity can complicate the provision of restorative treatment and the treatment of periodontal tissues [3, 4].

Dentine sensitivity is related to the exposure of dentine tubules resulting from either loss of tooth enamel or loss of periodontal tissue (gingival recession) [5, 6]. For dentine sensitivity to be incited, dentine tubules must be open at the dentine surface as well as remaining patent to the dental pulp [6, 7]. At the microscopic level dentine sensitivity is currently still described by the hydrodynamic theory proposed by Brannstrom [8, 9], which states that following dentine exposure to stimuli such as physical touch, temperature alteration, sweet liquid, and acidic liquid, changes in intratubular fluid movement and intratubular pressure occur. These changes can cause activation of intratubular nerve fibres of pulpal origin; this nerve excitation causes pain to be experienced [8, 10].

To manage dentine sensitivity products have been developed for at home use functioning primarily through two modalities: suppressing the excitability of intratubular nerve fibres or reducing the patency of exposed dentine tubules [2, 11, 12]. When acting to suppress the excitability of nerve fibres within dentine tubules, the most common approach is to elevate the extracellular potassium concentration within the dental pulp. This can be achieved through a patient applying a dentifrice containing potassium ions in a relatively soluble form to exposed dentine. Movement of potassium ions through patent dentinal tubules will act to raise the response threshold of pulpal nociceptors and reduce their ability to fire when provoked [13, 14].

When considering the effectiveness of agents that reduce dentine sensitivity through reduction in the patency of exposed dentine tubules, the most successful outcomes have been achieved through application of fluoride based gels to dentine [2, 11, 12]. It is well documented that application of dentifrices containing stannous fluoride [15–17] and sodium fluoride [18–21] can promote the deposition of mineral precipitates within open dentinal tubules, thereby reducing fluid flow in dentine tubules following exposure to stimuli. A dentifrice containing functionalised tricalcium phosphate and 950 ppm sodium fluoride has been shown to enable dentine tubule occlusion* in vitro*; application of this dentifrice to extracted bovine teeth following pH cycling was observed to reduce both dentine tubule opening and tubule diameter in comparison to pretreatment levels [22]. This finding is notable, as not only there is limited data demonstrating that ≤1000 ppmF dentifrices can effectively reduce dentine sensitivity, but the availability of such a dentifrice could lower the cost and complexity of “at home” treatment undertaken by a patient suffering from dentine sensitivity, a dentifrice containing ≤1000 ppmF being appropriate for the control of dental caries in low caries risk patients [23, 24]. Importantly, while assessment of the anticaries benefits of this dentifrice has been reported [25], no assessment of the efficacy of this dentifrice to reduce dentine sensitivity* in vivo* has taken place.

The aim of the present study was therefore to assess the effectiveness of a dentifrice containing 950 ppm fluoride and functionalised tricalcium phosphate (fTCP) in the reduction of dentine sensitivity* in vivo*. The assessment was undertaken as a parallel group, blinded randomised control trial. The null hypothesis was that the effectiveness of the dentifrice containing fTCP and 950 ppm fluoride would not be different to the effectiveness of a dentifrice containing 1000 ppm fluoride in reducing dentine sensitivity.

## 2. Materials and Methods

The present study was a single centre, parallel group, blinded (subjects and examiners) randomised controlled clinical trial conducted at the Westmead Centre for Oral Health, Westmead Hospital, Sydney, Australia. Ethical approval from the Western Sydney Local Health Network Human Research Ethics Committee was obtained prior to commencement of the study (SAC2010/11/4.6 (3223) HREC/10/WMEAD/202) and the trial was registered with the Australia New Zealand Clinical Trials Register. Subjects included within the study were from the pool of patients eligible to receive treatment at the Westmead Centre for Oral Health. From this patient pool, during an initial “off waiting list” examination, over 2500 individuals were asked if they experience dentine sensitivity. Eight hundred and fifty individuals indicated they suffered from dentine hypersensitivity and were willing to participate in the study. Of these 850 individuals a total of 71 subjects were recruited for the study having met the strict inclusion criteria. All subjects which participated in the study consented to participation prior to the inclusion. As part of the consent process, patients were made aware of adverse effects of dentifrices, namely, abrasion and staining of hard and soft tissues and the detrimental sequelae of excessive fluoride ingestion.

### 2.1. Inclusion and Exclusion Criteria

To be considered for this study, subjects were required to meet all of the following* inclusion* criteria:

1. being aged between 18 and 70 years;
2. demonstrating good general health with no history of chronic illness;
3. possession of at least 2 teeth with an exposed root surface which are responsive on probing (50 g force) or a 1 s duration cold air blast (70 psi); these teeth* were not to exhibit* diagnosed caries, defective restorations, or signs of fracture on initial assessment; these teeth* were not to have had* dental restorations, periodontal surgery, or orthodontics that has resulted in postoperative pain in the immediate past 3 months;
4. a willingness to read, understand, and sign the consent form;
5. a capability and willingness to brush teeth at least 2 times a day for 2 minutes on each occasion.

A subject was* excluded* from participation in the study if* any* of the following conditions applied:

1. use of a desensitising agent in the 3 months prior to the study;
2. undertaking regular medical treatment involving anti-inflammatory or analgesic use;
3. being pregnant or nursing;
4. exhibiting a known allergy to any ingredients in the examined dentifrices;
5. suffering from conditions which could increase the level of acid within the oral cavity: bulimia, gastric reflux disease;
6. excessive dietary exposure to acids; Lemons ≥ 2 times per day, raw tomatoes ≥ 2 times per day, acidic drinks ≥ 1 litre per day (sports drinks, energy drinks, or fruit juice), wine ≥ 3 standard glasses per day;
7. an inability to read the oral hygiene instructions provided to each participant.

### 2.2. Study Groups

Allocation of subjects to four study groups was randomised through use of a computer algorithm to limit the impact of age, gender, diet, and current level of oral hygiene on the study results. The four study groups (Groups A–D) were as follows.

*Group A.* This group brushed teeth twice daily with a dentifrice containing 1000 ppm fluoride ions (MFP): Colgate Cavity Protection (Colgate-Palmolive, New York, NY, USA,). Group A functioned as a negative control.

*Group B.* This group brushed teeth twice daily with a dentifrice containing 1000 ppm fluoride ions (NaF) + 19300 ppm Potassium ions (KNO_3_): Sensodyne Total Care (GlaxoSmithKline, Sydney, NSW, Australia). Group B functioned as a positive control.

*Group C.* This group brushed teeth twice daily with a dentifrice containing fTCP and 950 ppm fluoride ions (NaF): Clinpro Tooth Crème (3M ESPE, St. Paul, MN, USA).

*Group D.* This group brushed teeth twice daily with a dentifrice containing fTCP and 950 ppm fluoride ions (NaF): Clinpro Tooth Crème (3M ESPE, St. Paul, MN, USA) and a directed “pea sized” topical application of Clinpro Tooth Crème onto sensitive teeth before sleeping without rinsing.

At no time during the trial were subjects made aware of what test group they belonged to or which product they were using; all packaging was discarded prior to distribution and toothpaste tubes were wrapped in generic sticky white labelling.

At the study commencement, all subjects were provided with a new toothbrush and dental floss and were given the same oral hygiene instructions verbally and as a take home pamphlet. Subjects were also provided with a two-minute timer in a bid to maintain strict and consistent adherence to the prescribed two-minute brushing time twice daily.

### 2.3. Clinical Assessment

Patients were assessed at three time points during the study: baseline, 6 weeks, and 10 weeks. The clinical assessment was completed by three senior dental officers at Westmead Centre for Oral Health to limit the effect of clinician variation on study results. Standardisation was also maintained through each clinician recalibrating the process of stimulus provision to sensitive surfaces at the commencement of each assessment day. Examiners remained blinded to test groups to which participants belonged at all examination points throughout the study.

At each assessment, each sensitive tooth surface was exposed to three different stimuli that were applied directly onto the identified sensitive tooth. The three stimuli included an air blast, tactile stimulation, and application of a hypertonic solution. Each stimulus was applied according to the following standardised process.

*Air Blast.* The identified tooth surface was exposed to air delivered from a standard dental unit triplex syringe from an operating distance of approximately 1 cm for a period of 1 s at an operating temperature of 21°C (±5°C). A pressure gauge was mounted to the dental unit and calibrated prior to each assessment day to ensure air was delivered at a standard pressure of 70 psi.

*Tactile.* The identified tooth surface was stroked for 3 s using a standard dental explorer probe that was held perpendicular to the surface using a force of 50 g.

*Hypertonic Solution.* A 70% hypertonic sucrose solution was applied to the identified tooth surface for 3 s. The solution was at room temperature at the time of application.

**Figure 1:**
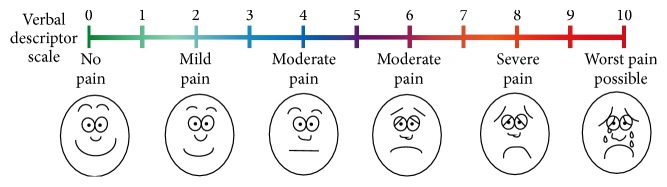
11-point Pain Numbered Rating Scale (NRS-11).

Following application of each stimulus patients rated the pain/sensitivity experienced using an 11-point numbered pain rating scale (NRS-11; Figure 1). These scores were recorded on clinical test forms.

Following the 6-week examination patients returned the study dentifrice they were issued and reverted to prestudy dentifrice use and habits. This allowed for a “wash-out” period to elapse before reexamination at 10 weeks to quantify any prolonged actions of each test dentifrice; 6-week usage of a dentifrice has been shown to provide sufficient time to allow maximum benefit of a desensitising product [26].

Patient compliance in the use of the study dentifrice was established at the 6-week examination. Patients were asked to return products which were issued and each tube was weighed to calculate the total amount of toothpaste used over the 6-week period.

### 2.4. Statistical Analysis

For each test group the mean pain score for each stimulus modality (air blast, tactile, and hypertonic solution) was calculated by patient and by tooth at each assessment point (baseline, 6 weeks, and 10 weeks). Additionally, the mean pain scores for each test group for each stimulus were combined and divided by 3 to give a total combined modalities sensitivity (CMS) score at each assessment point. The percentage change relative to baseline in mean pain score for each stimulus and the percentage change in CMS relative to baseline were calculated for each test group. ANCOVA (with baseline as the covariate) with a Tukey HSD post hoc test was used for between group comparisons.

## 3. Results

### 3.1. Baseline Summary

Of the 850 individuals from the Westmead Centre for Oral Health patient pool who indicated suffering from dentine sensitivity, 80 individuals satisfied the inclusion criteria. These 80 individuals were randomly allocated to the four study groups (A–D). The study population exhibited a mean age of 40.9 and a range of 17–67 years of age. Seventy-one of the 80 subjects completed the 10-week clinical study and complied with the protocol given. Of the participants who completed the study, there were 54 females and 17 males. No adverse soft tissue or hard tissue effects were observed by the assessing clinicians during the study; however one participant withdrew from the study reporting an allergic reaction to Colgate Cavity Protection. The 9 patients that did not complete the study did so as they no longer wanted to attend the recall appointments on the basis of convenience.

### 3.2. Evaporative (Cold) Sensitivity

**Figure 2:**
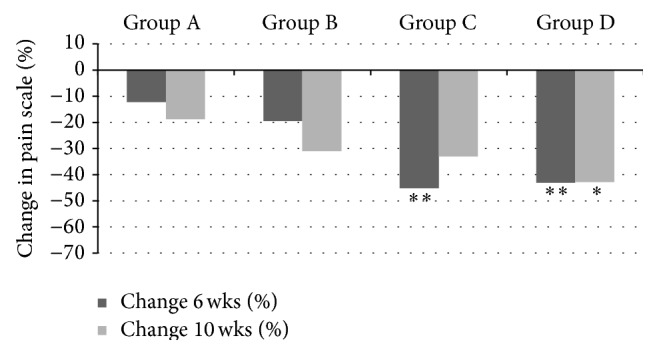
Percent change in Evaporative (cold) sensitivity scores from baseline to 6 weeks (cessation of treatment) and 10 weeks (4 weeks after treatment cessation). Group A: Colgate Cavity Protection (negative control); Group B: Sensodyne Total Care (positive control); Group C: Clinpro Tooth Crème (brushing only); Group D: Clinpro Tooth Crème (brushing and topical application). ∗ Scores significantly different from negative control (*P* < 0.05, 95% CI). ∗∗ Scores significantly different from both positive and negative control (*P* < 0.05, 95% CI).

At 6 weeks (end of the treatment phase) all groups showed a reduction in evaporative sensitivity score from baseline, with Colgate Cavity Protection showing a 12% reduction, Sensodyne Total Care a 19% reduction, Clinpro Tooth Crème (brushing only) a 45% reduction, and Clinpro Tooth Crème (brushing + topical application) showing a 43% reduction. The reduction in evaporative sensitivity scores from base line of both Clinpro Tooth Crème groups at 6 weeks was significantly greater (*P* ≤ 0.05, 95% CI) than the reduction in evaporative sensitivity scores of both the positive control group (Sensodyne Total Care) and the negative control group (Colgate Cavity Protection). There was no significant difference (*P* ≥ 0.05, 95% CI) in the reduction of evaporative sensitivity scores from baseline when comparing the Sensodyne Total Care and Colgate Cavity Protection groups at the end of the 6-week treatment phase (Tables 1 and 2 and Figure 2).

At 10 weeks (four weeks after cessation of treatment), all four groups demonstrated a reduction in evaporative sensitivity score from baseline, with Colgate Cavity Protection showing a 18% reduction, Sensodyne Total Care a 40% reduction, Clinpro Tooth Crème (brushing only) a 24% reduction, and Clinpro Tooth Crème (brushing + topical application) showing a 54% reduction. The reduction in evaporative sensitivity scores at 10 weeks for Clinpro Tooth Crème (brushing + topical application) and Sensodyne Total Care was significantly greater (*P* ≤ 0.05, 95% CI) than the reduction in evaporative sensitivity demonstrated by the negative control Colgate Cavity Protection. There was no significant difference (*P* ≥ 0.05, 95% CI) in the reduction in evaporative sensitivity scores at 10 weeks from baseline when comparing groups using Sensodyne Total Care, Clinpro Tooth Crème (brushing only), and Clinpro Tooth Crème (brushing + topical application).

### 3.3. Tactile Sensitivity

**Figure 3:**
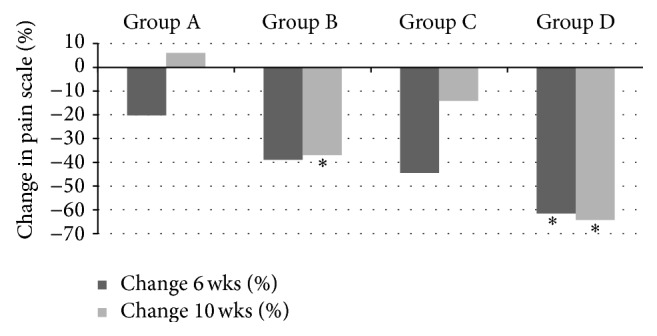
Percent change in tactile sensitivity scores from baseline to 6 weeks (cessation of treatment) and 10 weeks (4 weeks after treatment cessation). Group A: Colgate Cavity Protection (negative control); Group B: Sensodyne Total Care (positive control); Group C: Clinpro Tooth Crème (brushing only); Group D: Clinpro Tooth Crème (brushing and topical application). ∗ Scores significantly different from negative control (*P* < 0.05, 95% CI). ∗∗ Scores significantly different from both positive and negative control (*P* < 0.05, 95% CI).

At 6 weeks (end of the treatment phase) all groups showed a reduction in tactile sensitivity from baseline, with Colgate Cavity Protection showing a 20% reduction, Sensodyne Total Care a 39% reduction, Clinpro Tooth Crème (brushing only) a 44% reduction, and Clinpro Tooth Crème (brushing + topical application) a 62% reduction. At 6 weeks the only group to show a significant reduction (*P* ≤ 0.05, 95% CI) in tactile sensitivity scores in comparison to the negative control group (Colgate Total Protection) was Clinpro Tooth Crème (brushing + topical application). There were no other comparisons that displayed significantly different sensitivity scores; both Clinpro Tooth Crème groups were not significantly different (*P* ≥ 0.05, 95% CI) from the positive control group, Sensodyne Total Care (Tables 1 and 2 and Figure 3).

At 10 weeks (four weeks after cessation of treatment), not all groups showed a reduction in tactile sensitivity from baseline, with Colgate Cavity Protection showing a 6% increase in sensitivity. However, Sensodyne Total Care showed a 37% reduction in tactile sensitivity, Clinpro Tooth Crème (brushing only) a 14% reduction, and Clinpro Tooth Crème (brushing + topical application) a 64% reduction from baseline. A significant reduction (*P* ≤ 0.05, 95% CI) in tactile sensitivity at 10 weeks from baseline for Clinpro Tooth Crème (brushing + topical application) and Sensodyne Total Care in comparison to Colgate Cavity Protection was observed. No significant difference (*P* ≥ 0.05, 95% CI) in the reduction in tactile sensitivity scores from baseline to 10 weeks was observed between Clinpro Tooth Crème (brushing only) and Sensodyne Total Care. Additionally, no significant difference (*P* ≥ 0.05, 95% CI) in tactile sensitivity score reduction at 10 weeks was observed between Clinpro Tooth Crème (brushing only) and Colgate Cavity Protection (Tables 1 and 2 and Figure 3).

### 3.4. Hypertonic (Sweet) Sensitivity

**Figure 4:**
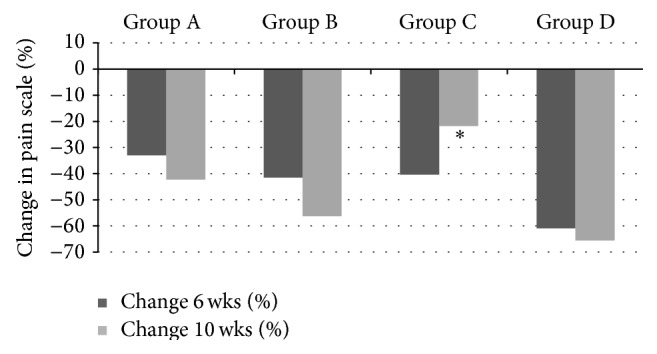
Percent change in hypertonic (sweet) sensitivity scores from baseline to 6 weeks (cessation of treatment) and 10 weeks (4 weeks after treatment cessation). Group A: Colgate Cavity Protection (negative control); Group B: Sensodyne Total Care (positive control); Group C: Clinpro Tooth Crème (brushing only); Group D: Clinpro Tooth Crème (brushing and topical application). ∗ Scores significantly different from negative control (*P* < 0.05, 95% CI). ∗∗ Scores significantly different from both positive and negative control (*P* < 0.05, 95% CI).

At 6 weeks all groups exhibited a reduction in hypertonic sensitivity from baseline, with Colgate Cavity Protection showing a 32% reduction, Sensodyne Total Care a 41% reduction, Clinpro Tooth Crème (brushing only) a 40% reduction, and Clinpro Tooth Crème (brushing + topical application) showing a 61% reduction. At 6 weeks the difference in % reduction from baseline was not significant (*P* ≥ 0.05, 95% CI) for any of the four groups (Tables 1 and 2 and Figure 4).

At 10 weeks, all groups exhibited a reduction in hypertonic sensitivity from baseline, with Colgate Cavity Protection showing a 42% reduction, Sensodyne Total Care a 56% reduction, Clinpro Tooth Crème (brushing only) a 22% reduction, and Clinpro Tooth Crème (brushing + topical application) showing a 66% reduction. The only groups to exhibit a significant difference (*P* ≤ 0.05, 95% CI) in hypertonic sensitivity reduction at 10 weeks compared to baseline were Sensodyne Total Care and Clinpro Tooth Crème (brushing + topical application). There was no significant difference (*P* ≥ 0.05, 95% CI) in hypertonic sensitivity reduction at 10 weeks between the groups using Colgate Cavity Protection, Sensodyne Total Care, and Clinpro Tooth Crème (brushing + topical application) (Tables 1 and 2 and Figure 4).

### 3.5. Combined Modalities Sensitivity (CMS)

**Figure 5:**
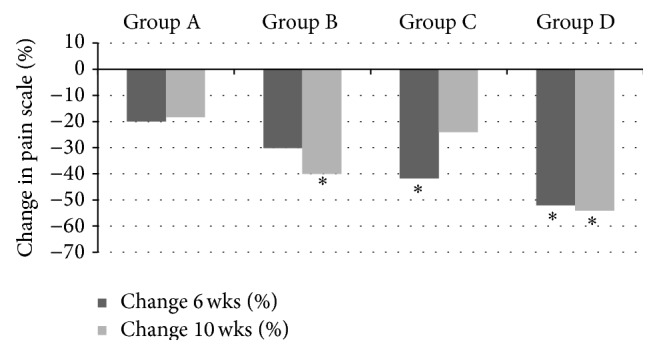
Percent change in combined modalities sensitivity scores from baseline to 6 weeks (cessation of treatment) and 10 weeks (4 weeks after treatment cessation). Group A: Colgate Cavity Protection (negative control); Group B: Sensodyne Total Care (positive control); Group C: Clinpro Tooth Crème (brushing only); Group D: Clinpro Tooth Crème (brushing and topical application). ∗ Scores significantly different from negative control (*P* < 0.05, 95% CI). ∗∗ Scores significantly different from both positive and negative control (*P* < 0.05, 95% CI).

At 6 weeks (end of the treatment phase) all groups exhibited a reduction in the combined sensitivity score (CMS) from baseline, with Colgate Cavity Protection showing a 20% reduction, Sensodyne Total Care a 30% reduction, Clinpro Tooth Crème (brushing only) a 42%, and Clinpro Tooth Crème (brushing + topical application) showing a 52% reduction. At the end of the 6-week treatment phase, both Clinpro Tooth Crème study groups showed a significant reduction (*P* ≤ 0.05, 95% CI) in CMS when compared to the negative control group using Colgate Cavity Protection. Clinpro Tooth Crème when used with an additional topical application also demonstrated a significant reduction (*P* ≤ 0.05, 95% CI) in dentine sensitivity compared to the positive control, Sensodyne Total Care, after 6 weeks (Tables 1 and 2 and Figure 5).

At 10 weeks (4 weeks after cessation of treatment), all groups showed a reduction in combined sensitivity score (CMS) from baseline, with Colgate Cavity Protection showing an 18% reduction, Sensodyne Total Care a 40% reduction, Clinpro Tooth Crème (brushing only) a 24% reduction, and Clinpro Tooth Crème (brushing + topical application) a 54% reduction. The reduction in CMS for Clinpro Tooth Crème (brushing + topical application) and Sensodyne Total Care was significantly greater (*P* ≤ 0.05, 95% CI) than the reduction in CMS from baseline of the negative control Colgate Cavity Protection at four weeks after cessation of treatment. There was no significant difference (*P* ≥ 0.05, 95% CI) in CMS reduction from baseline to 10 weeks between groups using Sensodyne Total Care, Clinpro Tooth Crème (brushing only), and Clinpro Tooth Crème (brushing + topical application) (Tables 1 and 2 and Figure 5).

The results of the present study indicate that the null hypothesis was rejected.

## 4. Discussion

The ability for fluoride ions sourced from a dentifrice to form fluoride-calcium precipitates that occlude dentine tubules with the consequence of reducing dentine sensitivity is well documented [18–21, 27, 28]. This ability occurs due to the negative electric charge of fluoride ions, which results in their binding with calcium cations. Once bound to calcium cations present within a dentifrice the fluoride ions are rendered incapable of combining with cations present at the tooth surface.

To overcome the effect of reduced fluoride ion availability at the tooth surface caused by intradentifrice fluoride-calcium bonding, dentifrice manufacturers have traditionally acted to increase the concentration of fluoride ions within a dentifrice [29]. In recent times, however, altering the chemical structure of calcium complexes within a dentifrice to reduce the bonding affinity between calcium cations and fluoride anions has been undertaken as an alternative solution to simple fluoride ion concentration increase. One such example of this has been the development and incorporation of altered calcium phosphate complexes [30], such as functionalised tricalcium phosphate, which is currently incorporated within Clinpro Tooth Crème.

Functionalised tricalcium phosphate (fTCP) is produced through beta tricalcium phosphate (*β*TCP) complexes being milled with sodium lauryl sulfate (SLS) [30]. The *β*TCP crystal structure exhibits several reactive sites including calcium-oxygen clusters (CaO_3_, CaO_7_, and CaO_8_) and lattice defects [31]. These reactive sites are available to undergo chemical interaction with anions such as fluoride ions. To reduce the reactivity of these sites within b-TCP complexes, the anionic surfactant SLS is added for incorporation within these reactive sites. The incorporation of SLS within the *β*TCP structure therefore impedes fluoride ions from combining with calcium ions in the dentifrice, so in turn potentially increasing the concentration of both calcium and fluoride to tooth surfaces [30].

Significantly Karlinsey et al. [25, 32–34] have reported a synergistic relationship between fTCP and fluoride with regard to enamel remineralisation; enamel that is remineralised through a fluoride/fTCP combination demonstrates significantly greater surface and subsurface rehardening following pH cycling compared to that achieved by fluoride application alone. Notably, the results of the present study suggest that this remineralisation synergy between fluoride and fTCP may also produce a benefit in terms of enhancing dentine tubule occlusion; the two test groups that utilised Clinpro Tooth Crème (950 ppm F) demonstrated a greater level of sensitivity relief for all stimuli except the hypertonic test at 10 weeks (group C) in comparison to the group using Colgate Total, despite the fact that Colgate Total contains a greater concentration of fluoride ions (1000 ppm F). Previous microscopic analysis showing the ability of Clinpro Tooth Crème to reduce the diameter of tubule openings following pH cycling to a greater degree than Sensodyne NUPRO 5000 and Topex Renew supports this possibility [22].

Within the present study a greater reduction in dentine sensitivity was observed when an additional topical application of Clinpro Tooth Crème was placed to supplement application provided through brushing alone. This finding is in contrast to reports in the literature stating that there is no evidence to suggest that additional topical application increases the effectiveness of a desensitizing dentifrice [1]. There are several reasons that might account for the greater reduction in sensitivity. First an intentional topical application of a dentifrice potentially allows a greater concentration of fluoride ions to be applied to a tooth surface in comparison to that applied through “unintentional” brushing alone. Secondly, through an additional topical application, fluoride ions can remain at a sensitive surface for a longer duration than following brushing and rinsing and thirdly through increasing the duration of fluoride ion presence upon a sensitive surface, the propensity for fluoride migration and tubule occlusion is raised.

The greater success of Clinpro Tooth Crème when applied as an additional topical application rather than brushing alone may also be a result of greater salivary fluoride concentration as a by-product of topical application and no rinsing following application. This possibility is consistent with studies that have examined the effect of patient activity following dentifrice application on the level of salivary fluoride [35]. Nordström and Birkhed determined that a topical application of dentifrice to a tooth surface combined with regular brushing resulted in a greater concentration of salivary fluoride than when compared to brushing alone [36]. Sjögren and Melin identified that a single post-brushing rinse with fluoridated water decreased fluoride concentration by a factor of two when compared to no rinsing, while rinsing with fluoridated water two times following brushing decreased salivary concentration by a factor of 5 when compared to no rinsing [37].

The reduction in CMS of the negative control group over the study duration can be in part attributed to the placebo effect which is well documented in dentine sensitivity studies [7, 38, 39]. Additionally the Hawthorne effect, which describes a positive response to noninterventional treatment, should also be assumed to have had some impact on the study results. Improved oral hygiene in patients enrolled in the study could have also reduced perceived dentine sensitivity across all groups as a reduction in tooth surface plaque facilitated increased dentifrice access to dentine tubules. These reasons may also account for the antihypersensitivity effects continuing for the month following the cessation of the use of the treatment dentifrices.

An identified limitation of the study was the numbers of patients enrolled in each study group. Due to the breadth of the potential patient pool, patients eligible for treatment at Westmead Centre for Oral Health, it was anticipated that including 40 subjects per group would be an achievable goal over a 3-year study period. As a result of the very strict exclusion criteria, especially the requirement that the use of a desensitising agent in the 3 months prior to the study excluded an individual from being a subject, patient recruitment was extremely difficult. However despite the smaller numbers than originally forecast per group, the results of the present study provide useful information. Not only are the numbers sufficient to demonstrate statistical significance, but when tooth number rather than patient number is used for analysis the subject number exceeds 50 for each group and the statistical outcomes remain the same.

## 5. Conclusions

The addition of fTCP to a dentifrice suitable for daily oral hygiene can enhance the ability of dentifrice fluoride to reduce dentine sensitivity. In the present study twice daily brushing with Clinpro Tooth Crème resulted in a similar reduction in dentine sensitivity to that achieved through brushing with a dentifrice containing KNO_3_ + F^−^ (Sensodyne Total Care). If Clinpro Tooth Crème is used twice daily for brushing in combination with a nightly topical application, it can be more effective in reducing dentine sensitivity than twice daily brushing with Sensodyne Total Care.

## Figures and Tables

**Table 1:** Study population and baseline sensitivity summary.

	Number of participants	Baseline sensitivity means (±SD)				
		Mean age	Combined modalities sensitivity score	Evaporative (cold) sensitivity	Tactile sensitivity	Hypertonic (sweet) sensitivity
Group A	20	45	2.93 (±2.30)	4.14 (±2.84)	2.50 (±3.02)	2.17 (±2.89)
Group B	17	39	3.09 (±1.92)	4.89 (±2.25)	1.95 (±2.55)	2.42 (±2.72)
Group C	16	39	2.28 (±1.53)	3.79 (±2.26)	1.63 (±2.31)	1.40 (±2.08)
Group D	18	40	2.89 (±2.22)	4.49 (±2.70)	1.86 (±2.65)	2.33 (±2.83)

Group A: Colgate Cavity Protection; Group B: Sensodyne Total Care; Group C: Clinpro Tooth Crème (brushing only); Group D: Clinpro Tooth Crème (brushing and topical application).

**Table 2:** Summary of changes in pain score (NRS-11) from baseline within each test group.

	6-week score (Tx cessation)		10-week score (4 weeks after Tx cessation)	
	Pain score adjusted means	% reduction from baseline	Pain score adjusted means	% reduction from baseline
Group A				
Evaporative	3.82	12%	3.50	19%
Tactile	1.60	26%	2.14	−6.10%
Hypertonic	1.42	33%	1.22	42%
CMS	2.26	20%	2.30	18%
Group B				
Evaporative	3.51	19%	2.97	31%
Tactile	1.22	40%	1.27	37%
Hypertonic	1.24	41%	0.92	56%
CMS	1.97	30%	1.69	40%
Group C				
Evaporative	2.39	45%	2.89	33%
Tactile	1.11	32%	1.74	14%
Hypertonic	1.26	40%	1.65	22%
CMS	1.65	42%	2.14	24%
Group D				
Evaporative	2.48	43%	2.46	43%
Tactile	0.77	62%	0.72	64%
Hypertonic	0.83	61%	0.73	66%
CMS	1.35	52%	1.29	54%

Group A: Colgate Cavity Protection; Group B: Sensodyne Total Care; Group C: Clinpro Tooth Crème (brushing only); Group D: Clinpro Tooth Crème (brushing and topical application). A positive value of percentage change indicates an improvement in sensitivity at the time of assessment compared to baseline.

CMS: combined modalities score is the average of all testing modalities, evaporative, tactile, and hypertonic.

Adjusted baseline means from ANCOVA: evaporative = 4.35; tactile = 2.00; hypertonic = 2.12; CMS = 2.83.
